# A Novel Test for Independence Derived from an Exact Distribution of *i*th Nearest Neighbours

**DOI:** 10.1371/journal.pone.0107955

**Published:** 2014-10-02

**Authors:** Sebastian Dümcke, Ulrich Mansmann, Achim Tresch

**Affiliations:** 1 Institute for Genetics, Universität zu Köln, Cologne, Germany; 2 Max Planck Institute for Plant Breeding Research, Cologne, Germany; 3 IBE, Ludwig-Maximilians-Universität, Munich, Germany; University of Bonn, Bonn-Aachen International Center for IT, Germany

## Abstract

Dependence measures and tests for independence have recently attracted a lot of attention, because they are the cornerstone of algorithms for network inference in probabilistic graphical models. Pearson's product moment correlation coefficient is still by far the most widely used statistic yet it is largely constrained to detecting linear relationships. In this work we provide an exact formula for the 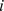
th nearest neighbor distance distribution of rank-transformed data. Based on that, we propose two novel tests for independence. An implementation of these tests, together with a general benchmark framework for independence testing, are freely available as a CRAN software package (http://cran.r-project.org/web/packages/knnIndep). In this paper we have benchmarked Pearson's correlation, Hoeffding's 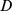, dcor, Kraskov's estimator for mutual information, maximal information criterion and our two tests. We conclude that no particular method is generally superior to all other methods. However, dcor and Hoeffding's 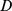 are the most powerful tests for many different types of dependence.

## Introduction

Dependence measures and tests for independence have recently attracted a lot of attention, because they are the cornerstone of algorithms for network inference in probabilistic graphical models. Pearson's product moment correlation coefficient is still by far the most widely used statistic in areas such as economy, biology and the social sciences. Yet Pearson's correlation is largely constrained to detecting linear relationships. Spearman [1] and Kendall [2] extended Pearson's work to monotonic dependencies. In 1948, Hoeffding [3] proposed a non parametric test for independence that is suited for many different functional relationships. Székely et al. [4] introduced the distance correlation (dcor) as a generalization of Pearson's correlation.

Other approaches build on mutual information (MI). MI characterizes independence in the sense that the MI of a joint distribution of two variables is zero if and only if these variables are independent. However, MI is difficult to estimate from finite samples. Kraskov et al. [5] proposed an accurate MI estimator derived from nearest neighbor distances. Reshef et al. [6] presented the maximal information coefficient (MIC), a measure of dependence for two-variable relationships which was heavily advertised [7] but lacks any statistical motivation.

dcor and Kraskov's estimator use the pair-wise distances of the points in a sample as a sufficient statistic. In this work we provide an exact formula for the 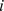
th nearest neighbor distance distribution of rank-transformed data (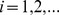). Based on that, we propose two novel tests for independence. An implementation of these tests, together with a general benchmark framework for independence testing, are freely available as a CRAN software package (http://cran.r-project.org/web/packages/knnIndep). In this paper we have benchmarked Pearson's correlation, Hoeffding's 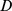, dcor, Kraskov's estimator for MI, MIC and our two tests. We conclude that no particular method is generally superior to all other methods. However, dcor and Hoeffding's 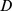 are the most powerful tests for many different types of dependence. Circular dependencies are best recognized by our tests. This type of dependence is fairly common, e.g., if two dependent periodic processes are monitored. An example from biology is the expression of a transcription factor and one of its target genes during the cell cycle [8].

## Exact distribution of the *i*th nearest neighbour distances

Consider a set of 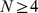 points that are distributed ‘randomly' on a surface. In what follows, we derive the distribution (conditional distribution) of the 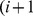)th nearest neighbor of a point (given the distance to its previous neighbors). We assume the points drawn from the following model: Let 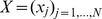 and 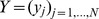 be permutations of the numbers 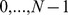 that are drawn uniformly from the set of all permutations of 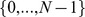. The points 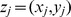, 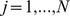, lie on a torus of size 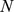 which is endowed with the maximum distance as a metric. I.e., the distance between two points is given by
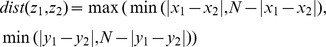



Fix a reference point 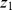. Let 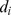, 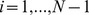 denote the distance of the 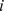-th nearest neighbor of 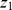 to 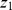 and 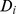 the random variable associated with it. Since this distance measure is translation invariant, let without loss 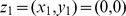. Importantly, all points 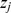 have pairwise different 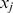 and 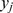. A point at distance 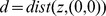 to the origin must have at least one of its coordinates equal to 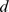 or 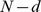. This implies that there are at most 4 points exactly at distance 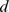 to the origin. Our target is the calculation of the joint probability of observing the whole sequence of nearest neighbor distances 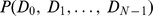, of the conditional probability 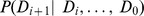 and the marginal 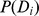. The main work will be the calculation of the probability 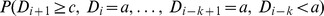 for given values 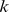, 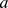 and 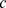. Once this is done, 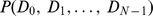, 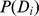 and 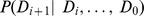 can be derived by elementary calculations (section S1 in Methods S1).

**Figure 1:**
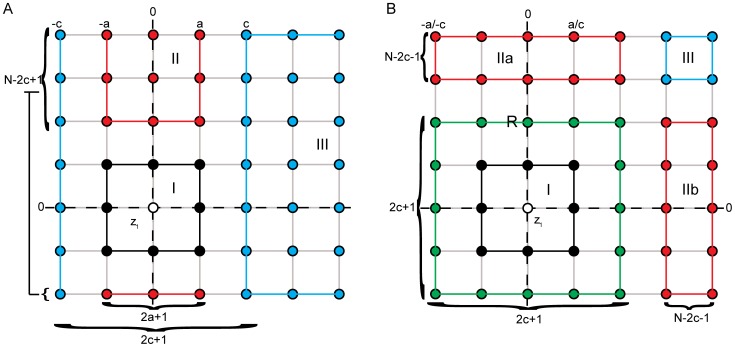
Diagrams explaining Equations 1 and 2 for 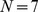, 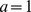 and 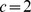 (panel A) and 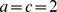 (panel B) with the reference point 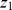 at coordinates 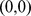. **A**: We define 3 regions I, II and III (black, red and blue points respectively). Region I has the least number of constraints and the number of admissible configurations is the number of possibilities to draw 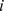 points from 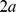 positions without replacement nor ordering: 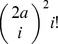. The number of admissible configurations for region II is given by the number of rows 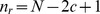 available and the number of columns which remain to be filled 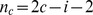 according to 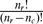. Region III has the remaining 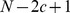 points freely distributed, yielding 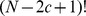 admissible configurations. **B**: In the case 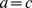 we add an additional region 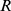 of 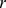 points exactly at distance 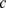 (green points). There can be 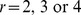 such points. Region I has size 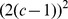 and 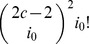 admissible configurations with 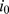 the number of points strictly inside the square of distance 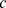. Region IIa and IIb are symmetric and handled analogous to region II in panel A with 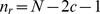 and 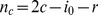. Region III has 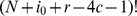 admissible configurations analogous to panel A.



First we determine 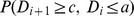 by counting the number of admissible point configurations and dividing through 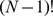, the number of all possible point configurations with 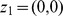 fixed. When counting configurations, we repeatedly exploit the fact that each horizontal and each vertical grid line contains exactly one point from the sample. In case of 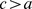, we split the torus into 3 regions (Figure 1). Region I is a square of side length 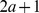. It contains 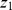 and 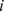 additional points at arbitrary positions. The number of possibilities to draw an 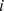-tuple from 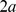 positions (recall that one position is already taken by 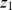) without replacement is 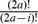. Thus, there are 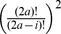 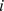-tuples describing an admissible configuration in region I. However, each configuration is counted 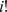 times, since the order of the points does not matter. Hence, the number of unique configurations in region I equals 
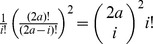
. For the second region we have 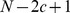 possible y-coordinates and 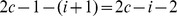 columns to be filled with sample points (note that the columns 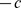 and 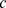 belong to region III and that 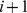 columns are already taken by points in region I). This yields 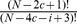 unique configurations for region II. There are 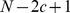 points remaining which can be placed freely in the remaining 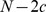 columns/rows, yielding 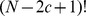 possibilities. Together we obtain:

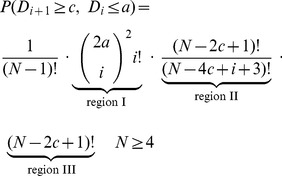
(1)In the case of 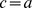 there is one more complication, because we have a region R of points exactly at distance 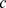, containing at least the 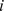-th and 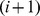-th neighbor of 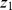, where the region I overlaps with regions IIa and IIb (Figure 1). Let 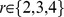 be the number of points in region R and 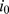 the number of points strictly inside the square of distance 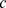. We derive a general formula for all admissible configurations in the case of 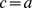, 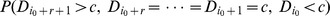. Denote by 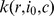 the number of admissible point configurations in region R (see section S2 in Methods S1 for a derivation of 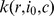). Table 1 lists all possible admissible combinations of points in region R. Counting the admissible configurations strictly inside regions I, IIa, IIb and III is similar to the above cases (Equation 1). This leads to the following general formula for all admissible configurations:

**Table 1:** Counts for points lying exactly on the border region R.

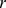	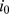	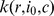 ; let 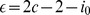	condition
1	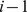	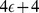	if 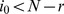
2	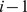	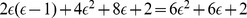	if 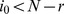
3	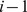 , 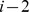	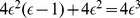	if 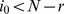
4	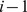 , 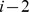 , 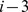	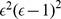	if 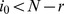

For each possible number of points 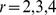 on the border region R and each possible number of points 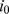 strictly inside of region I, we give the the number of admissible combinations of points in region R. The derivations of the number of admissible combinations is shown in the section S2 in Methods S1.



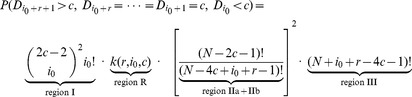
(2)The sum over all possible tuple 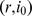 in Table 1 gives the probability 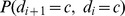 in the general case:
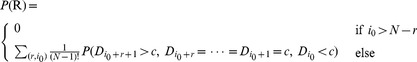
(3)


The above calculations only hold if region R is a genuine square, for large values of 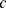 R degenerates to a pair of lines (one horizontal and one vertical line). These cases are covered in the extended formula
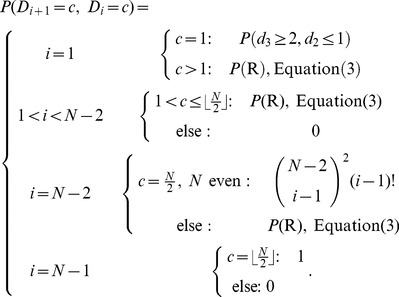
(4)


Analogously we can count the number of possible configurations where 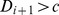, some 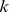 points 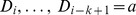 and all other points 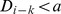 and deduce the following probability:
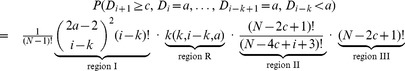
(5)


Since the above formulas involve tedious calculations, we validated the formulas for 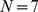 and 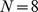 by counting the occurrence of each possible configuration among all 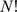 configurations. Additionally, we checked the validity of our formula for larger 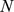 (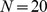) by taking 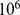 random configurations and comparing the empirical frequency 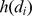 with 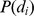 (section S3 in Methods S1).

**Figure 2:**
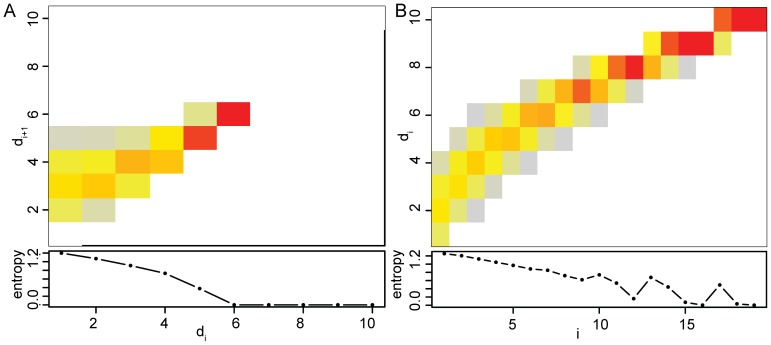
**A**: Conditional distribution 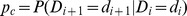 for 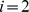, 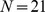 (*top*) and the entropy 
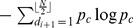
 (*bottom*). The probability 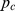 of observing large 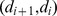 is zero for distances larger than 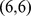 when 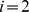. The lower triangle is empty because 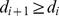 and the entropy is constantly decreasing for increasing values of 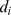 because the possible 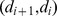 decrease towards 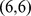. **B**: Marginal distribution 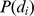 for 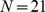 (*top*) and entropy 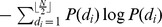 (*bottom*). With increasing 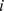, the distribution becomes narrower and the entropy tends towards 0, as the number of possible distances to the 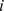
th nearest neighbor decrease. The non-monotonic behavior of the entropy for large values of 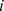 is due to downstream constraints imposed by the maximal distance 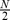. For testing independence, we advise using all 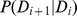 until the value of 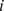 where the entropy starts increasing again (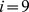 in this example).

Figure 2 shows the distribution of 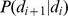 and 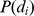. The conditional distribution is shown for 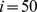. The marginal distribution is highly peaked with a low variance that decreases with increasing 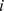 (and reaches 0 for 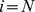).

The formulas have been implemented in the statistical language R [9] with emphasis on a numerically stable implementation as we deal with small numbers. The implementation is vectorized for speed. Still there is a computational penalty through the many factorials and logarithms that have to be calculated. For a sample of size 320, calculating all 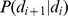 takes 4.1 seconds on a single workstation (single thread, Intel Core i5-2500 CPU @ 3.30GHz). Runtime for larger samples is shown in Figure S1 in File S1 and indicates a practical limit on the sample size of 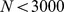 (which takes up to 3 minutes) and a complexity of 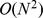.

For practical reasons, we assumed that the points lie on a torus (distances on the torus are translation-invariant and therefore our formulas for 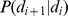 and 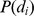 hold for all points in the sample). This will bias results when applied to points on a plane, as points on the border will have different nearest neighbors when projected on the torus. The bias is less pronounced for close neighbors (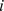 small), thus we limit our statistics to 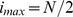. We do not expect to lose statistical power, since the information content of 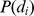 for large 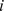 approaches zero (see Figure 2).

The derivation of 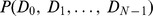, 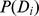 and 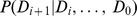 is based on Equations (1–5), see section S1 in Methods S1.

## Tests based on the *i*th nearest neighbour distribution

It has been shown that the distance of the 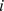
th nearest neighbour of some point 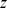 can be used to estimate the local (log) density at 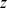 [5]. Our idea is to use the full sequence of nearest neighbour distances for assessing local density. For a sample point 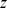, let 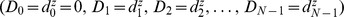 the sequence of neighbour distances. If 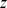 lies in a dense region, we expect this sequence to increase slower than in a region with lower density.

### Distributional tests

The sequence of nearest neighbor distances of a point 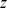, 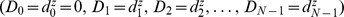 is a 4th order Markov chain, i.e.,
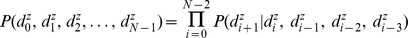



That way, taking 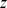 as the center point, the distances 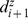, given the four previously observed distances 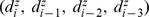, are pairwise independent for all 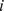. On the other hand this is not true for the distances 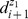 and 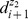
(not even if we condition the four previously observed distances). This follows from the triangle inequality in metric spaces, 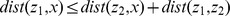, which implies that 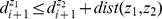.

Let the random variable 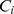 be defined by the process of drawing a point 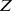 uniformly from 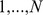 and then drawing 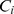 according to the distribution 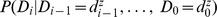. Let 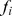 denote the probability function of 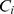, it is given by
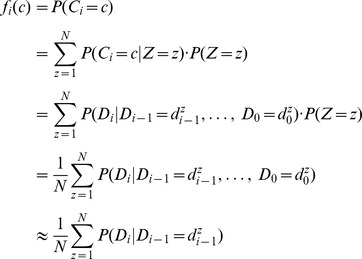



We consider the observed values 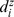, 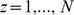, as (not necessarily independent) realizations of 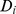. Their empirical frequency 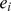 is
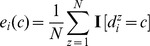
where 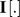 denotes the indicator function with values in 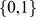. Pearson's 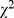 test [10] can be used to test for the fit of 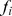 to 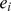:
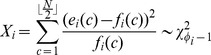


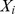
 is a 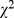-distributed test statistic with 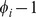 degrees of freedom where 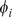 is the number distances 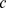 with 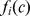 strictly positive. Our final test statistic is:
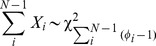



Alternatively the empirical and theoretical cumulative distributions corresponding to 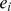 and 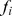 can be compared by an Anderson-Darling [11] or a Cramér-von Mises test, which proved inferior to Pearson's 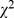 test (section S4 in Methods S1).

### Test for location

We have the idea to compare the distribution of the 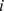
th neighbour distances observed in a sample with a suitable null distribution by means of their location. The most robust measures of location are mean or median, however in our studies of samples taken from joint distributions with low mutual information, we realized that many points do not show exceptional nearest neighbour distances. The difference to a sample drawn from independent 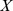 and 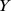 distributions was made up by few points that had extreme nearest neighbour distances. This lead us to use extreme values as a test for location. The pvalue of a two-sided test based on 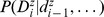 is 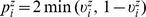, with 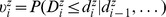. We summarize, for all 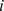
th neighbours, the 2-sided pvalues by their minimum
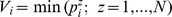



Our test statistic 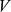 is obtained by aggregating the 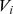 values: 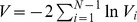.

## Construction of a benchmark set

Benchmarking was done on distributions 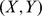 given by 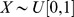, and 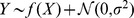. Here, 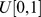 denotes a uniform distribution on the interval 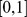, and 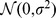 denotes a Gaussian distribution with mean 0 and variance 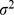. The function 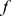 was chosen as one of the following: linear, quadratic, cubic, sine with period 0.5, circular, 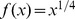 and a step function (see Figure S2 in File S1). This choice was inspired by a comment by Simon & Tibshirani (http://statweb.stanford.edu/tibs/reshef/script.R, [12]) to the publication of the method MIC by Reshef et al. [6]. The noise parameter 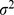 determines the degree of dependence between 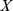 and 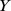, i.e., the mutual information 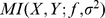. The latter was estimated using an approximation 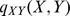 to the density 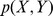 for which the mutual information can easily be calculated. We make 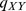 a piecewise-constant density on a sufficiently fine quadratic grid 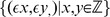 with 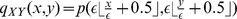. In our case, 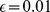 yielded sufficient precision. It is elementary to calculate the mutual information of 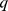 by
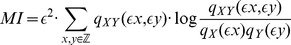



Here, 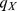 and 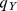 denote the marginal densities with respect to 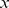 and 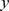.

To make the results comparable for different 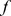, we fixed an MI value 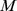 and chose 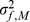 such that 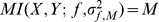. This was done for 20 MI values, 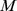 ranging from 0.01 to 0.5. The noise levels 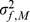 are listed in section S5 in Methods S1. Samples from all dependencies 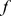 with 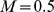 is shown in Figure S2 in File S1.

So far performance evaluation of measure of dependence was only done on functional dependencies. Here we introduce “patchwork copulas” as a new non-functional dependence of 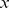 and 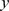. Fix a grid size 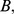 say 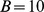. Our density 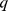 will be a piece-wise constant function defined on a rectangular 2D grid on the unit square (with uneven grid line spacing) such that its marginal distributions are uniform (i.e., we will define a copula). The parameters of our distribution are the values 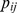, 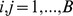, with 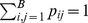. Let 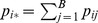 and 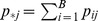. Let 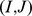 be a random variable which selects the grid rectangle 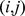 with probability 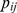, i.e., 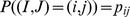, 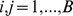. Our distribution 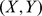 is then defined by 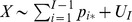, 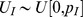, and 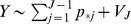, 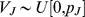. The density in the grid rectangle 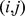 can be computed as 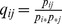. It is elementary to verify that the marginals of 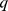 are uniform and that the mutual information of 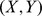 is
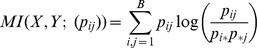



To generate samples with a desired MI value, we choose suitable values for 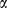 and 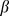. We draw i.i.d. samples 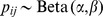, 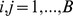, and then rescale the 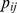 by dividing them by their sum. This process is repeated with different 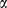, 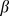 until 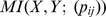 is close enough to the desired MI value. The resulting dependence resembles a patchwork quilt of dense and spread out point clouds (Figure S3 in File S1).

Typically the points are considered embedded in Euclidean spaces [5], however the distance function can easily be adapted to model the geometry of a torus. We benchmarked some methods on both geometries (Euclidean plane and torus) and found that all methods were sensitive to changes of geometry.

We made the benchmark framework publicly available under a GPL3.0+ license. It is implemented in R [9] and contains code for generating the dependence structures as well as plotting the results. An example is given in section S6 in Methods S1.

## Comparison of methods

**Figure 3:**
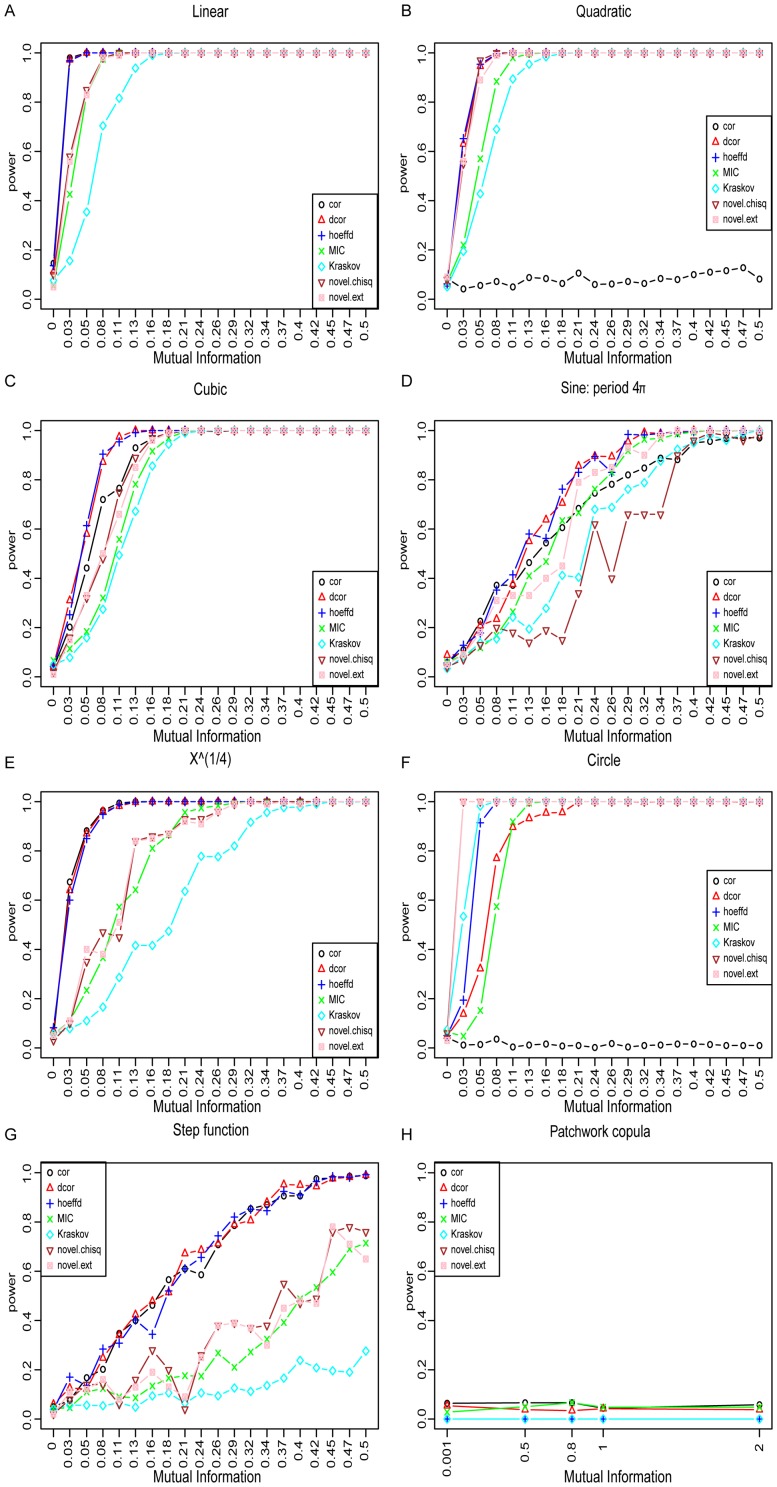
Benchmark of all methods. cor denotes Pearson's product moment correlation coefficient, dcor distance covariance, hoeffd Hoeffding's D, MIC denotes MIC, novelTest.chisq is our test based on Pearson's 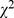 test and novelTest.ext is our test based on extreme paths. Each plot shows the power (on the y-axis) against the MI (x-axis). We examine 8 different types of dependence: linear, quadratic, cubic, sine with period 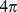, 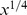, circle, step function and the dependence called "patchwork copula'' (**A–H**)

We compared both our tests (based on 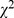 and extreme paths) to Pearson's product moment correlation coefficient, distance correlation (dcor, [4]), Hoeffding's D [3], Kraskov's estimator for mutual information [5] and MIC [6]. For each type of dependence and each given value of MI, we generated a test set of 500 samples each consisting of 320 points from the respective dependence type. Test statistics were calculated for each sample. Additionally we generated a reference set of 500 samples with 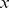 and 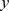 values drawn independently which is used to calculate the cutoff value corresponding to a significance level of 5%. The power of each method was estimated as the fraction of samples that were called significant according to the cutoff. Results are shown in Figure 3. Additionally we generated receiver operating curves (ROC) for each type of dependence and MI value (Figures S4–S9 in File S1).

The method of Hoeffding and dcor perform well throughout all types of dependence considered except for the circular dependence. Our methods have a performance that places them after dcor and Hoeffding's method and before MIC. In the case of the circular dependence, our methods perform best, achieving maximum power at mutual information of 0.03. We suspect that is due to the fact that a circle geometrically resembles two crossing lines when projected onto a torus (Figure S11 in File S1). To test this hypothesis we projected all types of dependence onto the torus and reran the whole benchmark (Figure S10 in File S1). We observe that the cubic, sine and step functions are not detected by any method, even at the same MI.

The scaling of the plots in Figure 3 to the MI of the underlying joint distribution, enables the direct visual comparison of different dependence types. On the one hand this reveals that some types of dependence seem to be more difficult to detect for all methods (step function, sine curve and the ''patchwork copula"). On the other hand each method performs best on different types of dependence.

**Figure 4:**
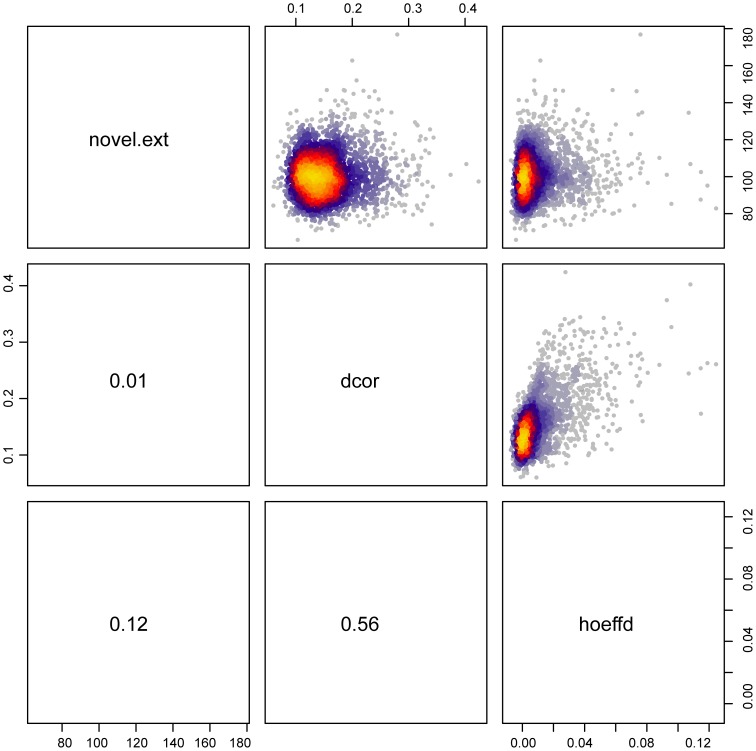
Performance on WHO data. novelTest.ext denotes our test based on extreme paths, dcor distance covariance and hoeffd Hoeffding's D. All methods were applied to all comparison between pairwise variables which had Pearson's product moment correlation coefficient near zero to exclude linear relationships. Only pairwise complete observations were used as most methods cannot handle missing vallues. All comparisons include ate least 81 datapoints. In total we compare all 3 methods on 2971 variable pairs.

We compared method of Hoeffding, dcor and our test based on extreme paths on a dataset from the World Health Organization and partner organizations. This dataset is available at http://www.exploredata.net/ftp/WHO.csv. We ran the methods on all pairwise comparisons that have a squared Pearson's product moment correlation coefficient lower than 0.001 to exclude any linear relationships. As most method cannot handle missing values, we further restricted the comparisons to have at least 81 pairwise complete observations. This leads to 2971 pairwise comparison shown in Figure 4. All test statistics are uncorrelated for the pairs in which no linear dependency was detected leading again to the conclusion that no method is uniformly more powerful.

## Discussion

We have derived an exact formula for the distribution of the distances of the 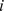 th nearest neighbour of a given point. This distribution assumes rank transformed bivariate data from two independent variables. While this result is of independent interest, we used it to construct two non-parametric tests of independence for bivariate data. Similar to Kraskov's estimator, our test statistic is purely based on nearest neighbour distances. In contrast to Kraskov's estimator which requires an arbitrarily fixed 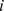, we simultaneously take into account the whole sequence of 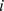
th nearest neighbours (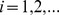). This improves on Kraskov's estimator, if used as a score for independence testing. Our tests use rank transformed data, because this is a prerequisite for applying the exact nearest neighbour distributions derived in this paper. The rank transformation is often used as a primary step to estimating mutual information, therefore we consider it an uncritical step in our procedure. Our tests perform almost as well as the best competitors dcor and Hoeffding's 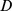 and they perform better than the recently proposed MIC statistic. We believe that the power of our method could be further improved in the Euclidean plane if our 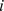
th neighbour statistic would be adapted to account for boundary effects in the Euclidean plane. Although our methods try to account for the dependence of the variables 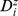, 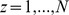, we necessarily lose power because their exact dependence structure is not known. Alternatively we propose to take all distances 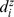 for a point 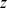 and apply a sequential testing approach for calling points that are located in dense regions. The number of these points could serve as a test statistic. The rationale is that under the null hypothesis of independence there should be fewer points 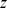 considered significant in the sequential test than for dependent samples.

Next we reviewed competing methods and presented a benchmark framework for performance testing on different types of dependence structures and topologies (Euclidean and toroidal). The benchmark framework and our novel tests for independence are publicly available as an R [9] package on CRAN (http://cran.r-project.org/web/packages/knnIndep). By scaling each type of dependence to a common set of mutual information values we allow comparison between all dependence types. Remarkably, when benchmarked on patchwork copulas, all methods fail. This is particularly intriguing for MIC as by design it should detect the grid structure of the data. In the case of the circular dependence, our methods perform best, while the method of Hoeffding and dcor perform well throughout all types of dependence considered. This in turn shows, that all tests we investigated are biased towards the detection of certain types of dependence structures.

## Supporting Information

Code S1 R code of the analysis of the WHO dataset.
(PDF)

File S1 Figures supporting results from the main text.
(PDF)

Methods S1 Supporting Methods for the main results.
(PDF)
