# Supplementary material for: A Novel Test for Independence Derived from an Exact Distribution of ith Nearest Neighbours
Source: PLoS One. 2014 Oct 2;9(10):e107955. doi: 10.1371/journal.pone.0107955 (PMC4183502; doi:10.1371/journal.pone.0107955)
Supplement: Code S1 — R code of the analysis of the WHO dataset. (PDF) [file pone.0107955.s001.pdf]

Supporting Code

September 1, 2014

```
data = read.csv("WHO.csv")
#remove non variables
data = data[,setdiff(colnames(data),c("Country","CountryID","Continent"))]
data = data[,-319] #column only contains NA values
#helper function to generate pairwise complete observations
pairwise.complete = function(x,y,rank=TRUE){
        rem = apply(cbind(x,y),1,function(i){any(is.na(i))})
        if(rank){
                return(list(x=rank(x[!rem],ties.method="random"),y=rank(y[!rem
        }else{
                return(list(x=x[!rem],y=y[!rem]))
        }
}

#get Pearson's correlation for all pairwise comparisons
comparisons = combn(1:ncol(data),2)
cors = apply(comparisons,2,function(col){
        a=pairwise.complete(data[,col[1]],data[,col[2]],rank=FALSE)
        return(c(cor(a$x,a$y),length(a$x)))
})
#take all pairwise comparisons that have a very low Pearson correlation
#and at least 81 pariwise complete observations
comp = comparisons[,cors[1,]^2 < .001 & cors[2,] > 80] #2971

#run our extreme value test
library(knnIndep)
ext.results = apply(comp,2,function(col){
        #only keep pairwise complete cases
        a = pairwise.complete(data[,col[1]],data[,col[2]],rank=FALSE)
        novelTest.extreme(a$x,a$y,20)
})

#run Hoeffding's D on the data
library(Hmisc)
hoeffd.pval = function(...){hoeffd(...)$P[1,2]}
```

```
hoeffd.results = apply(comp,2,function(col){
        a=pairwise.complete(data[,col[1]],data[,col[2]],rank=FALSE)
        hoeffd.pval(a$x,a$y)
})
#compare all 3 methods
library(LSD)
postscript(file="who_comp.ps",paper="special",width=7,height=7)
pairs(cbind("novel.ext"=ext.results,dcor=dcor.results,"hoeffd"=hoeffd.results
dev.off()
```
