# Supplementary material for: A Novel Test for Independence Derived from an Exact Distribution of ith Nearest Neighbours
Source: PLoS One. 2014 Oct 2;9(10):e107955. doi: 10.1371/journal.pone.0107955 (PMC4183502; doi:10.1371/journal.pone.0107955)
Supplement: File S1 — Figures supporting results from the main text. (PDF) [file pone.0107955.s002.pdf]

Supporting Figures

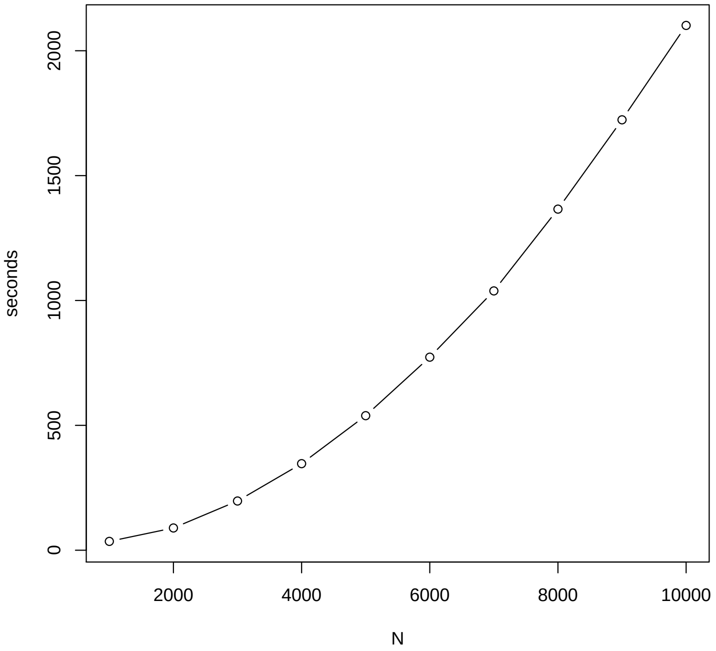

Figure 1: Runtime according to sample size N for calculating $P(D_i \mid D_{i-1})$ for all points and all nearest neighbors

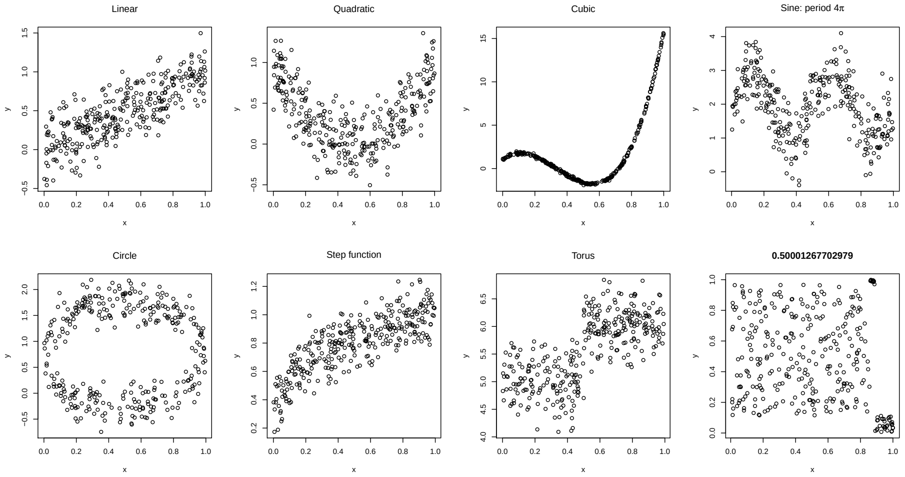

Figure 2: All considered functional dependencies with $MI = 0.5$

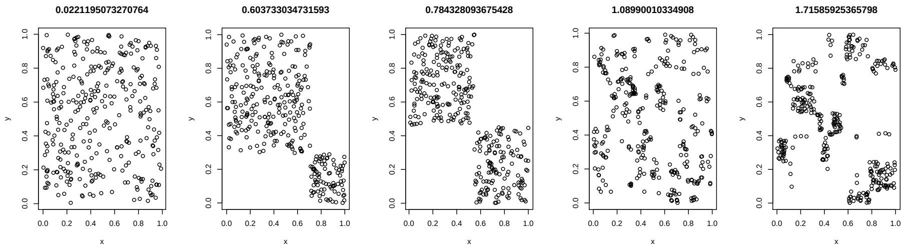

Figure 3: Scatterplots of the patchwork copula dependence for 320 points and 20×20 grid (parameters for Beta distribution: $(0.01, 1)$). This data is uniform in x and y but the joint distribution has a non-functional dependence. The mutual information is indicated above each plot.

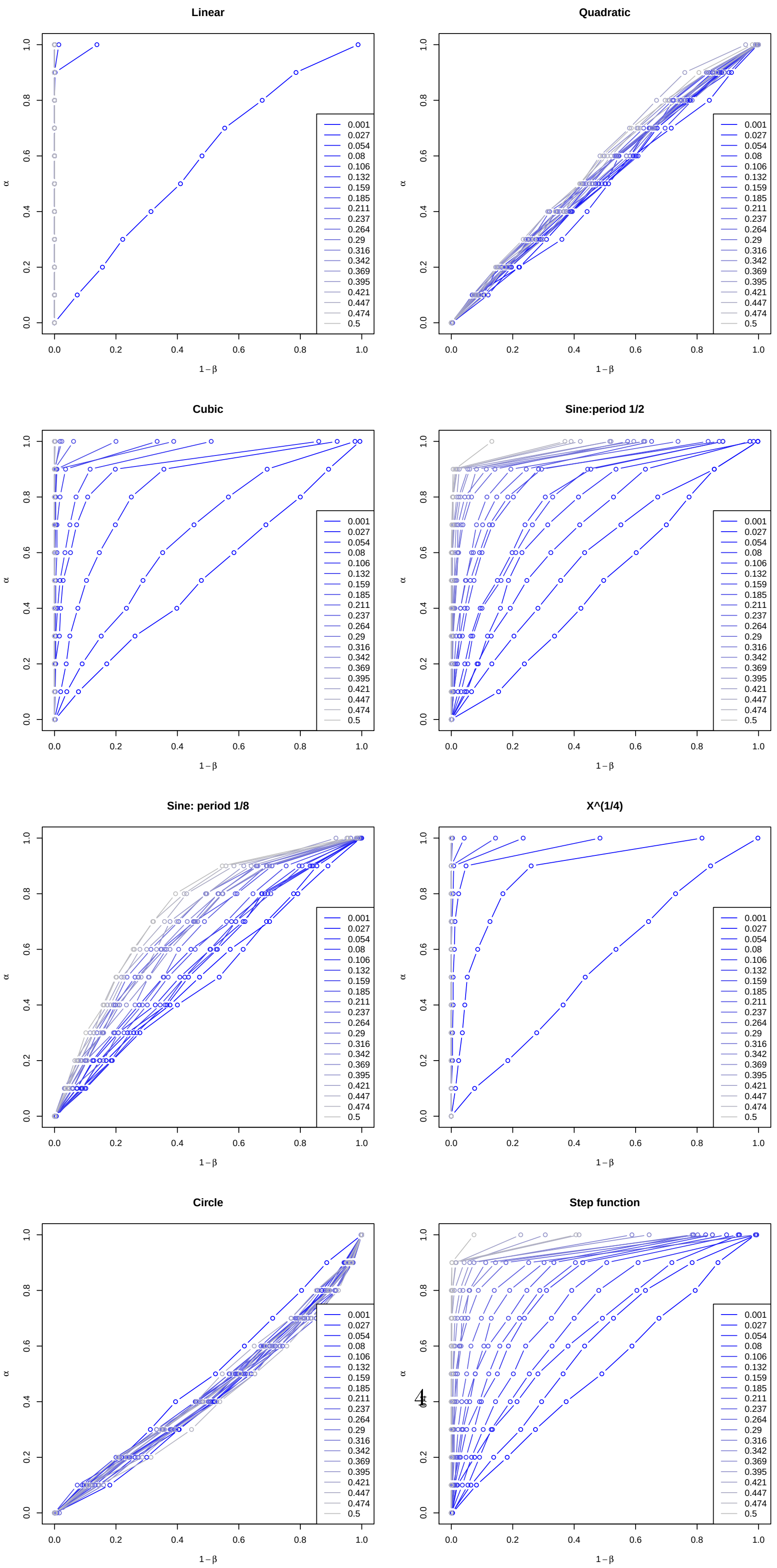

Figure 4: **Pearson's product moment correlation coefficient:** ROC curves

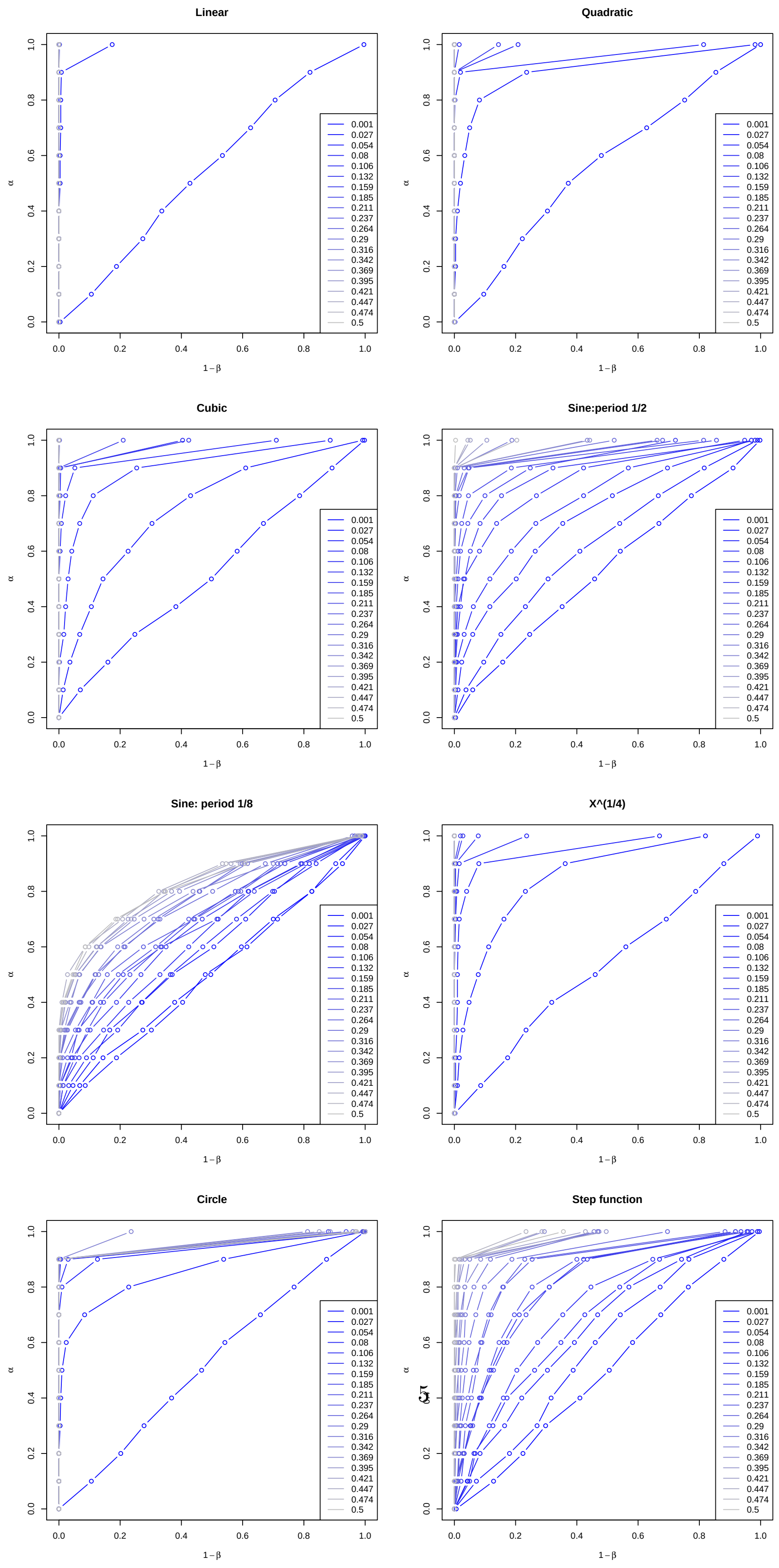

Figure 5: **dcor**: ROC curves for all 20 noise levels per functional dependency

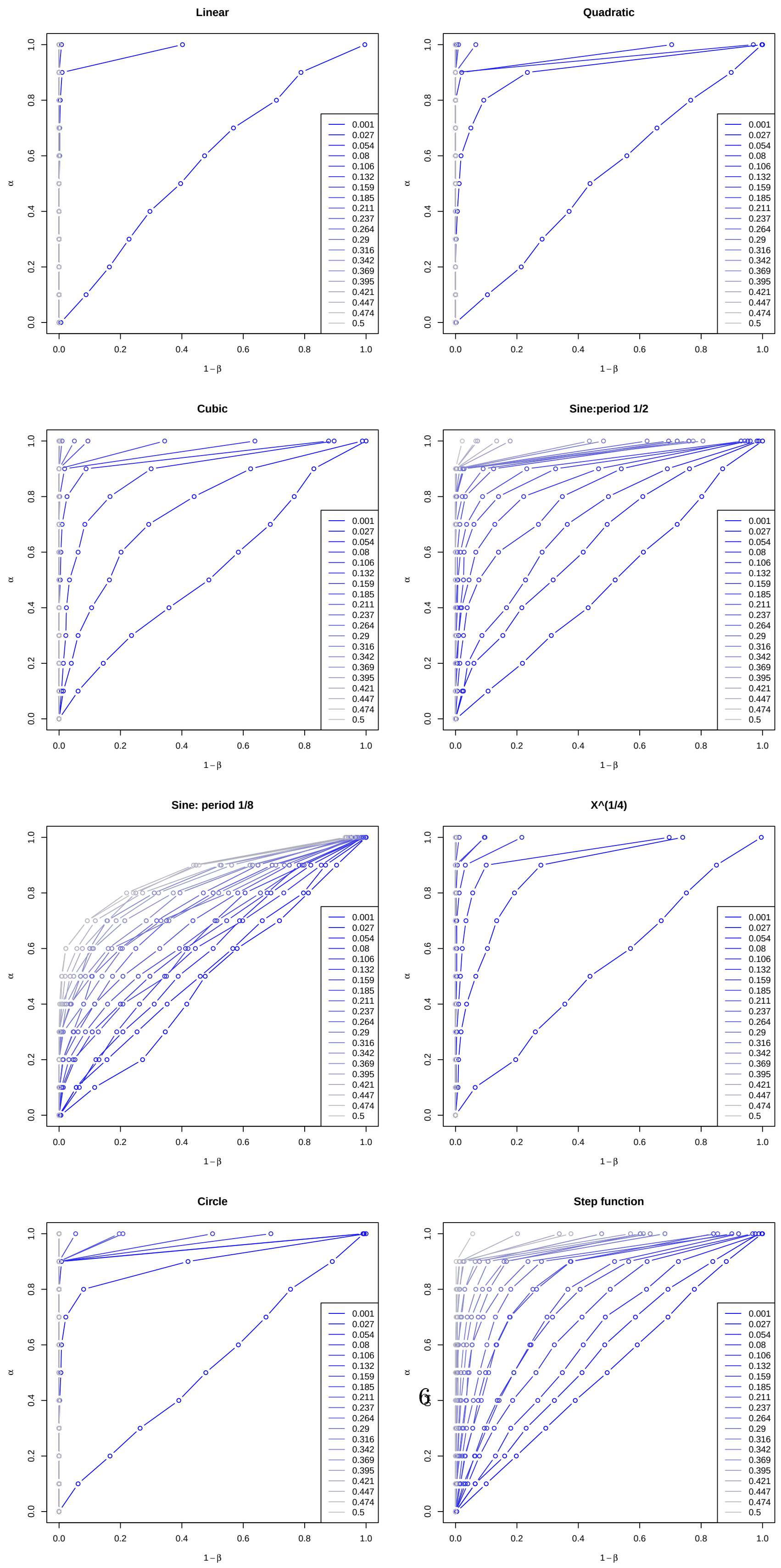

Figure 6: **Hoeffding's method:** ROC curves for all 20 noise levels per func-

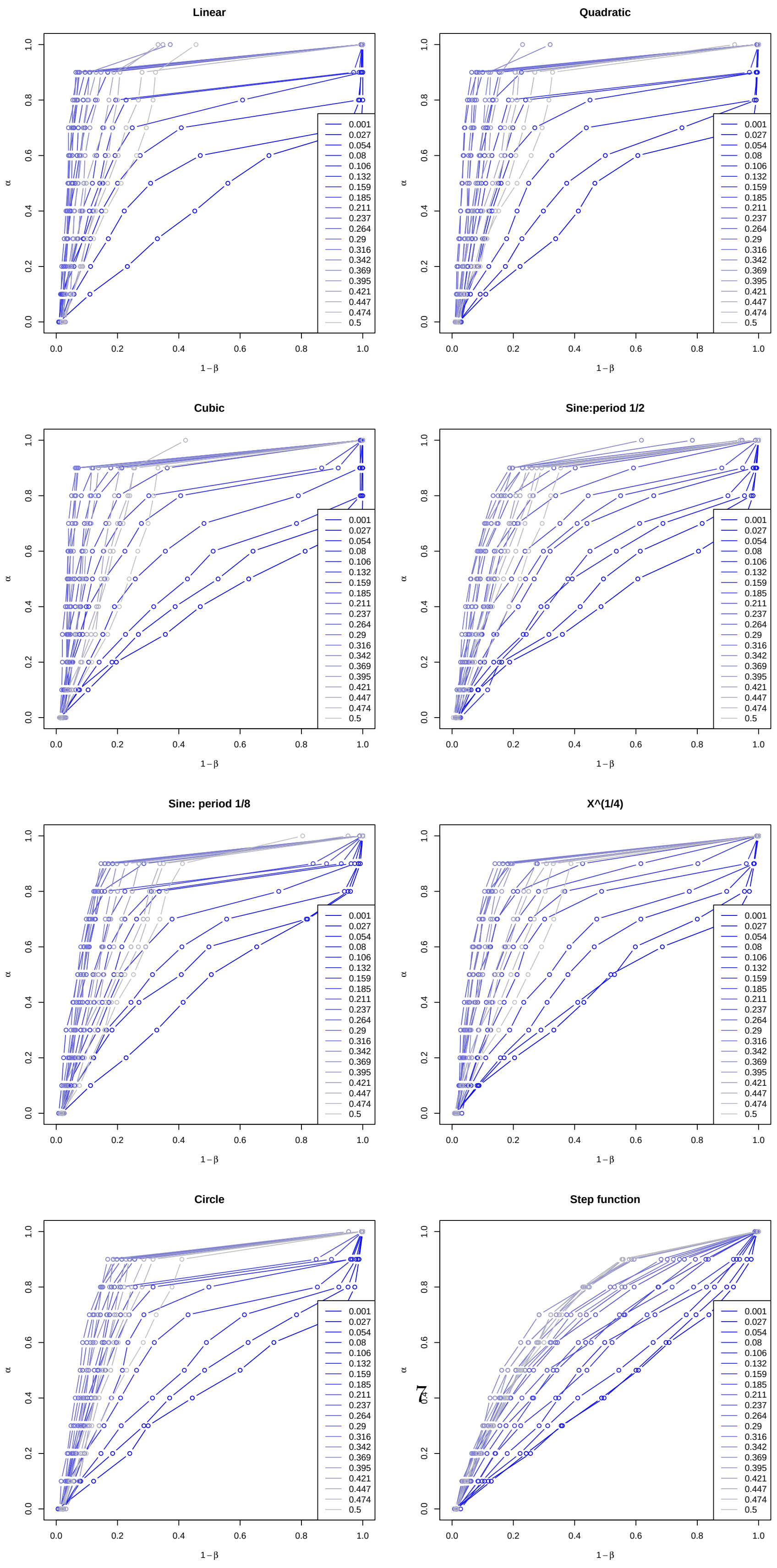

Figure 7: **MIC:** ROC curves for all 20 noise levels per functional dependency.

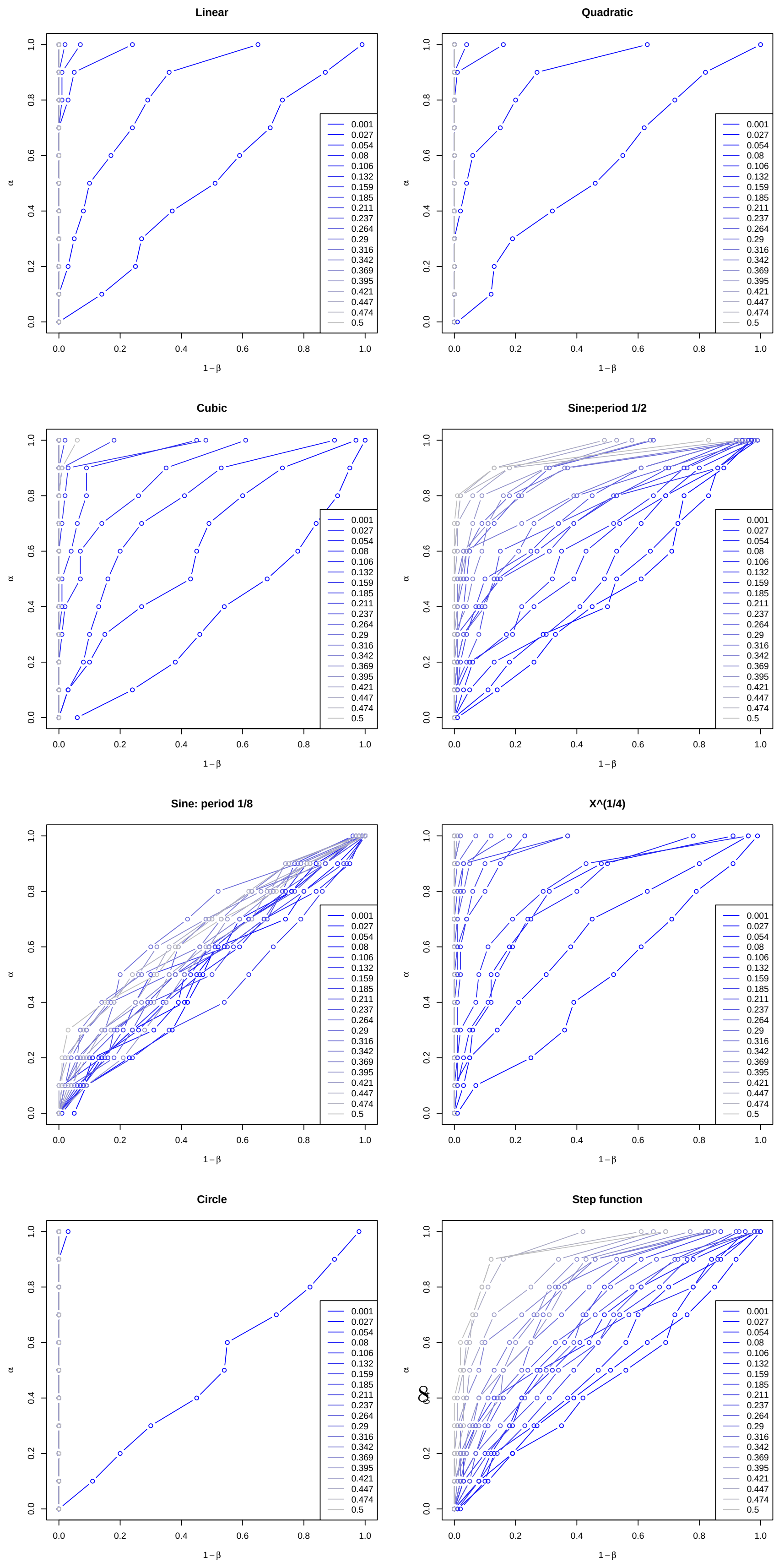

Figure 8: **Novel distributional test:** ROC curves for all 20 noise levels per

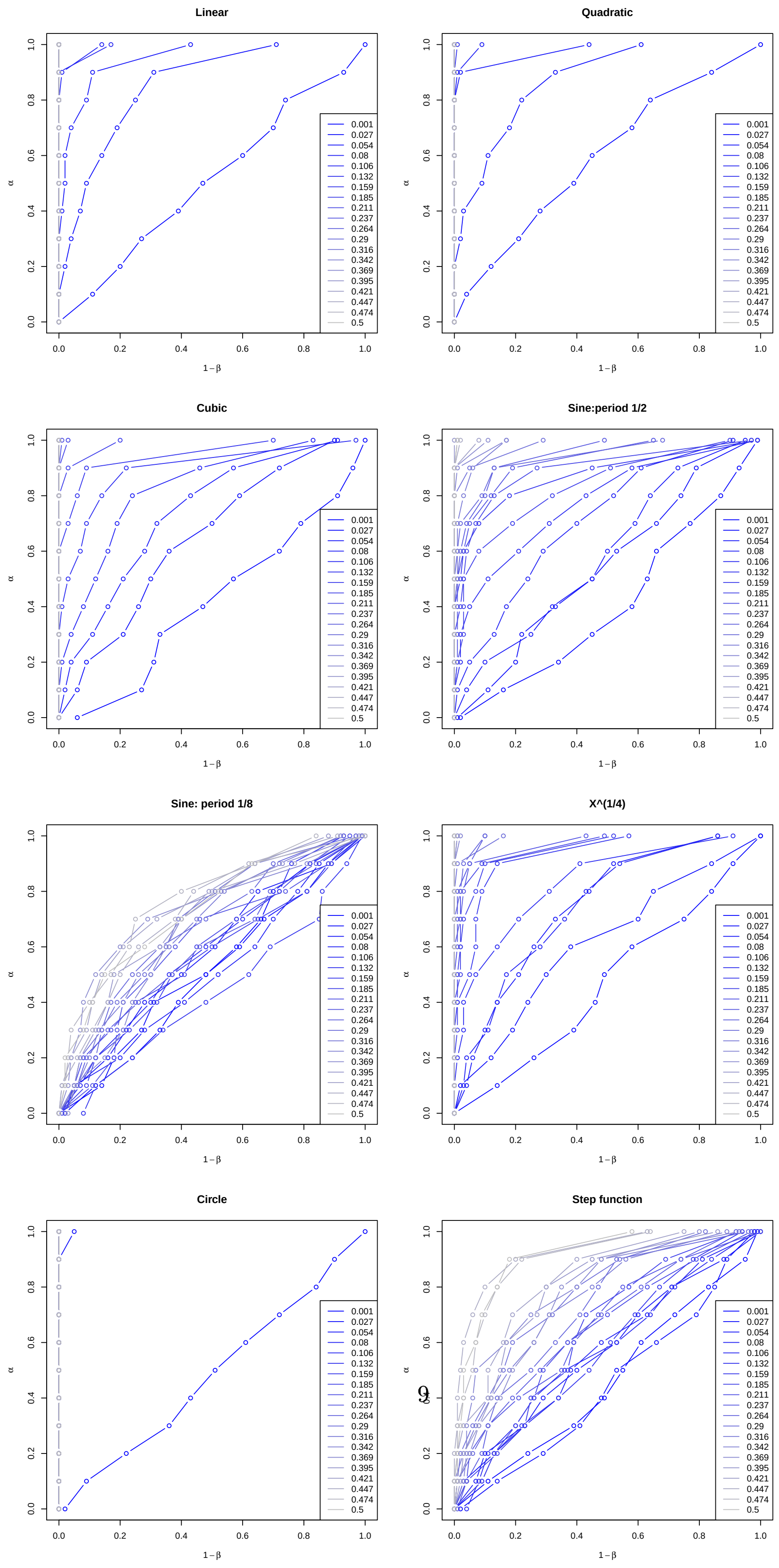

Figure 9: **Novel test for location:** ROC curves for all 20 noise levels per

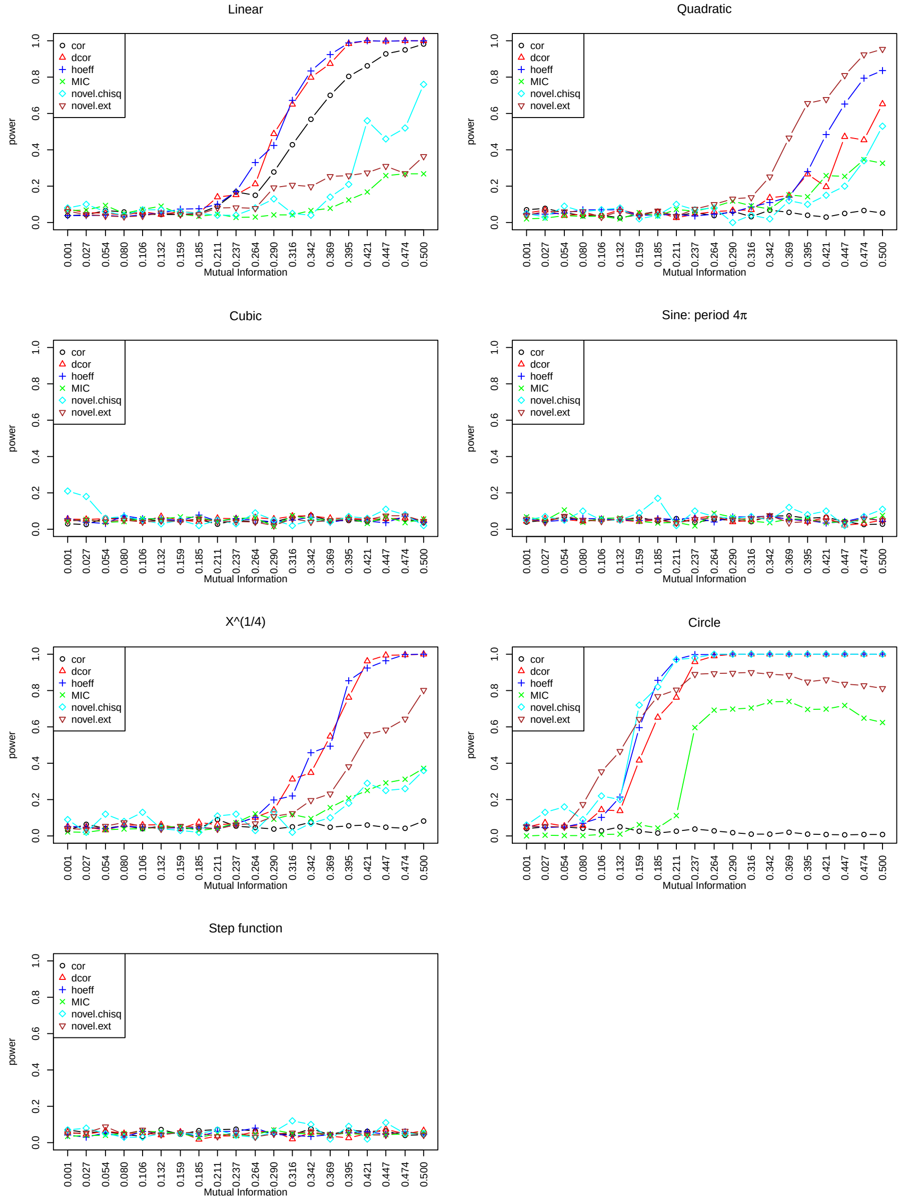

Figure 10: Benchmark of all methods on all functional dependence structures projected onto the torus. Cubic, sine and step function dependence are harder to detect on the torus even though the projection does not change to mutual information of the dependence. For the novel test only 100 samples were used to measure power, which explains the higher variance of these curves. Our novel test for location beats all other method on data with a quadratic functional dependence.

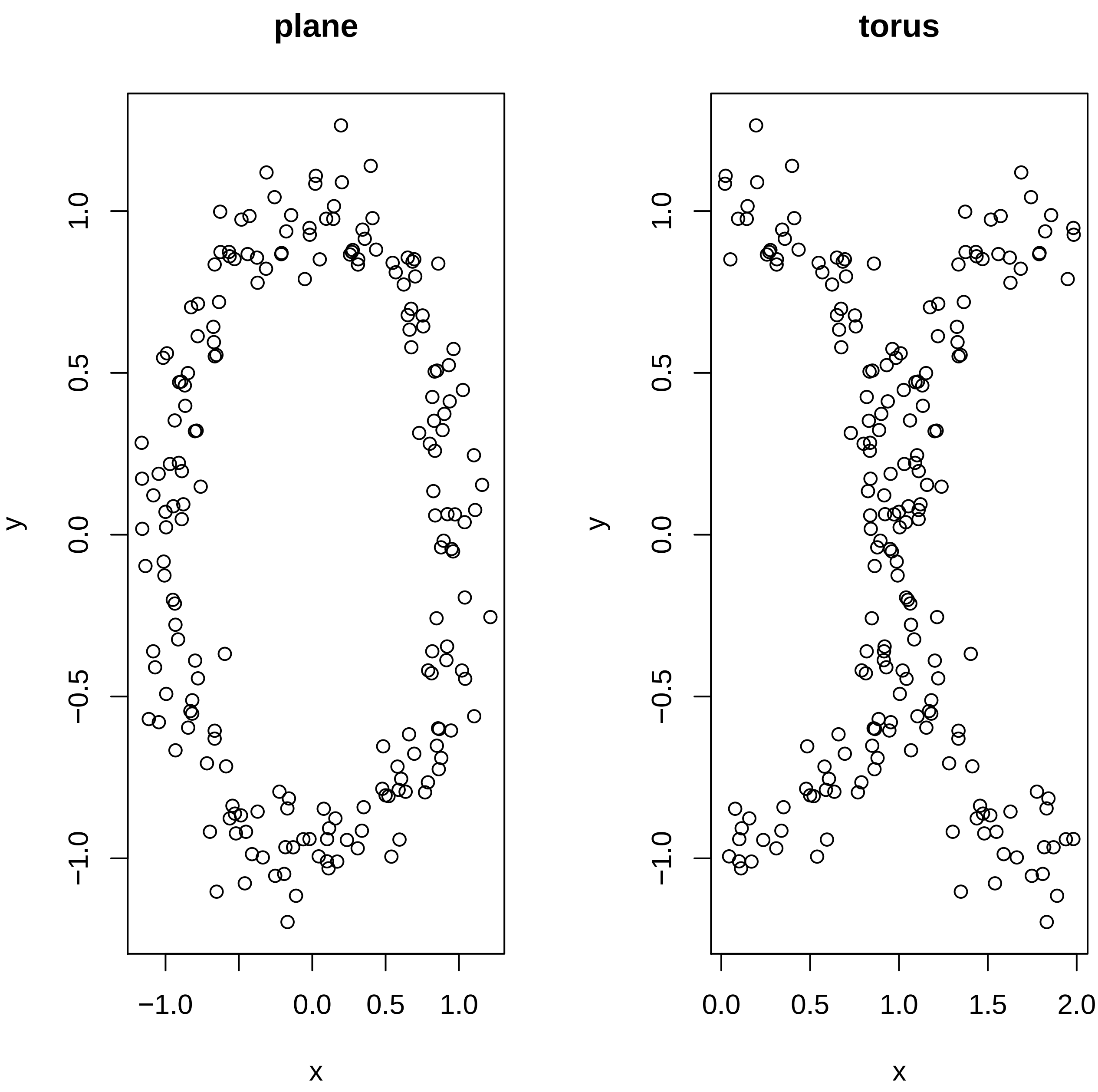

Figure 11: Projecting a circular dependence onto a torus resembles two crossed lines.
