# Supplementary material for: A Novel Test for Independence Derived from an Exact Distribution of ith Nearest Neighbours
Source: PLoS One. 2014 Oct 2;9(10):e107955. doi: 10.1371/journal.pone.0107955 (PMC4183502; doi:10.1371/journal.pone.0107955)
Supplement: Methods S1 — Supporting Methods for the main results. (PDF) [file pone.0107955.s003.pdf]

Supporting Methods

September 1, 2014

1 Derivation of $P(D_0,\, D_1, \ldots,\, D_{N-1})$ and $P(D_{i+1} \mid D_i)$

1.1 Derivation of $P(D_{i+1} \mid D_i)$ from $P(D_{i+1} \geq c,\, D_i \leq a)$

Set $d_0 = 0$ for convenience, and let $a \leq c$, $a \leq \lfloor \frac{N}{2} \rfloor$, $i \in \{0, ..., N-2\}$. Note that

$$P(D_{i+1} \geq c,\, D_i = a) = P(D_{i+1} \geq c,\, D_i \leq a) - P(D_{i+1} \geq c,\, D_i \leq a-1) \tag{1}$$

First we calculate the marginal distribution

$$\begin{aligned} P(D_i = a) &= P(D_{i+1} \geq a,\, D_i = a) \\ &= P(D_{i+1} \geq a+1,\, D_i = a) + P(D_{i+1} = a,\, D_i = a) \\ &= P(D_{i+1} \geq a+1,\, D_i \leq a) - P(D_{i+1} \geq a+1,\, D_i \leq a-1) + P(D_{i+1} = a,\, DS_i = a) \end{aligned} \tag{2}$$

The three terms in the last line are known from Equations 1 and 4 from the main text. Consequently,

$$\begin{aligned} P(D_{i+1} \leq c \mid D_i = a) &= 1 - P(D_{i+1} \geq c+1 \mid D_i = a) \\ &= 1 - \frac{P(D_{i+1} \geq c+1,\, D_i = a)}{P(D_i = a)} \end{aligned} \tag{3}$$

which then leads to

$$P(D_{i+1} = c \mid D_i = a) = P(D_{i+1} \leq c \mid D_i = a) - P(D_{i+1} \leq c-1 \mid D_i = a) \tag{4}$$

All these formulas of course only hold for those choices of a and i for which the probability $P(D_{i+1} \geq a,\, D_i = a)$ is non-zero.

1.2 Derivation of $P(D_{i+1} = c \mid D_i = \cdots = D_{i-k_2+1} = a, D_{i-k_2} < a)$ from $P(d_{i+1} \geq c, d_i = a, \ldots, d_{i-k+1} = a, d_{i-k} < a)$

By a simple induction,

$$\begin{aligned} & P(D_i = a, \ldots, D_{i-k+1} = a, D_{i-k} < a) \\ = \;& P(D_{i+1} > a, D_i = a, \ldots, D_{i-k+1} = a, D_{i-k} < a) + P(Dd_{i+1} = a, D_i = a, \ldots, D_{i-k+1} = a, D_{i-k} < a) \\ = \;& ... \\ = \;& \sum_{l=k}^{4} P(D_{i-k+l+1} > a, D_{i-k+l} = \cdots = D_{i-k+1} = a, D_{i-k} < a) \end{aligned} \tag{5}$$

The derivation of the central 'counting' task from the main text (Equation 2) together with the recursive formula (5) gives us:

$$\begin{aligned} & P(D_{i+1} = c \mid D_i = a, \ldots, D_{i-k+1} = a, D_{i-k} < a) \\ = \;& \frac{P(D_{i+1}=c,D_i=a,...,D_{i-k+1}=a,D_{i-k}<a)}{P(D_i=a,...,D_{i-k+1}=a,D_{i-k}<a)} \\ = \;& \frac{P(D_{i+1}\geq c,D_i=a,...,D_{i-k+1}=a,D_{i-k}<a)-P(D_{i+1}\geq c+1,D_i=a,...,D_{i-k+1}=a,D_{i-k}<a)}{P(D_i=a,...,D_{i-k+1}=a,D_{i-k}<a)} \\ \overset{(5)}{=} \;& \frac{P(D_{i+1}\geq c,D_i=a,...,D_{i-k+1}=a,D_{i-k}<a)-P(D_{i+1}\geq c+1,D_i=a,...,D_{i-k+1}=a,D_{i-k}<a)}{\sum_{l=k}^{4} P(D_{i-k+l}=\cdots=D_{i-k+1}=a,D_{i-k}<a,D_{i-k+l+1}>a)} \end{aligned} \tag{6}$$

1.3 Derivation of $P(D_{i+1} = c \mid D_i = c, \ldots, D_{i-k+1} = c, D_{i-k} < c)$ from $P(D_{i_0+1} = \cdots = D_{i_0+r} = c, D_{i_0} < c, D_{i_0+r+1} > c)$

Using the Equation (5),

$$\begin{aligned} & P(D_{i+1} = c \mid D_i = c, \ldots,\, D_{i-k+1} = c,\, D_{i-k} < c) \\ = & \tfrac{P(D_{i+1}=c,\, D_i=c, \ldots,\, D_{i-k+1}=c,\, D_{i-k}<c)}{P(D_i=c, \ldots,\, D_{i-k+1}=c,\, D_{i-k}<c)} \\ \overset{(5)}{=} & \tfrac{\sum_{l=k+1}^{4} P(D_{i-k+l}=\cdots=D_{i-k+1}=c, D_{i-k}<c, D_{i-k+l+1}>c)}{\sum_{l=k}^{4} P(D_{i-k+l}=\cdots=D_{i-k+1}=c, D_{i-k}<c, D_{i-k+l+1}>c)} \end{aligned}$$

1.4 Derivation of $P(D_{i+1} \mid D_i, \ldots,\, D_0)$

At most four points can be at the same distance to a reference point z. Therefore

$$P(D_{i+1} = c \mid D_i, \ldots, D_0) = \begin{cases} P(D_{i+1} = c \mid D_i = c, \ldots, D_{i-k_1+1} = c, D_{i-k_1} < c) & \text{if } D_{i+1} = D_i = c \\ P(D_{i+1} = c \mid D_i = \cdots = D_{i-k_2+1} = a, D_{i-k_2} < a) & \text{if } D_i = D_{i-1} = a,\, a < c \\ P(D_{i+1} \mid D_i, D_{i-1} < c) = P(D_{i+1} \mid D_i) & \text{if } D_{i+1} > D_i > D_{i-1} \end{cases}$$

where k_1 is the number of points that are at the same distance to z than the $(i+1)$th nearest neighbor (excluding the $(i+1)$th nearest neighbor) and k_2 is the number of points that are at the same distance to z than the ith nearest neighbor (including the ith nearest neighbor itself). $k_1 \in [0, 4]$, $k_2 \in [0, 4]$

1.5 Derivation of $P(D_0,\, D_1, \ldots,\, D_{N-1})$ from $P(D_{i+1} \mid D_i, \ldots,\, D_0)$

Let E_k the event that $d_k < d_{k+1}$. Let $d_{>k} = (d_{k+1}, ..., d_n)$ and $d_{\leq k} = (d_0, ..., d_{ik})$. By a counting argument,

$$P(d_{>k}, d_{\leq k} \mid E_k) = P(d_{>k} \mid E_k) \cdot P(d_{\leq k} \mid E_k) \tag{7}$$

Given some i, since at most 4 points can have the same distance to the origin, there is a largest $k \in \{i, i-1, i-2, i-3\}$ such that E_k holds. Then,

$$P(d_{i+1}, d_i, ..., d_{i-3} \mid E_k) \overset{(7)}{=} P(d_{i+1} \mid E_k) \cdot P(d_i, ..., d_{i-3} \mid E_k)$$

and

$$\begin{aligned} P(d_{i+1} \mid d_i, ..., d_0) &= P(d_{i+1} \mid d_i, ..., d_0, E_k) \\ &= \frac{P(d_{i+1}, d_i, ..., d_0 \mid E_k)}{P(d_i, ..., d_0 \mid E_k)} \\ &\overset{(7)}{=} \frac{P(d_{i+1}, ..., d_{k+1} \mid E_k) \cdot P(d_k, ..., d_0 \mid E_k)}{P(d_i, ..., d_{k+1} \mid E_k) \cdot P(d_k, ..., d_0 \mid E_k)} \\ &= \frac{P(d_{i+1}, ..., d_{k+1} \mid E_k) \cdot P(d_k, ..., d_{i-3} \mid E_k)}{P(d_i, ..., d_{k+1} \mid E_k) \cdot P(d_k, ..., d_{i-3} \mid E_k)} \\ &\overset{(7)}{=} \frac{P(d_{i+1}, ..., d_{i-3} \mid E_k)}{P(d_i, ..., d_{i-3} \mid E_k)} \\ &= P(d_{i+1} \mid d_i, ..., d_{i-3}) \end{aligned} \tag{8}$$

The probability of observing the complete sequence of nearest neighbors to a reference point factorizes into

$$\begin{aligned} P(D_0,\, D_1, \ldots,\, D_{N-1}) &= \prod_{i=0}^{N-2} P(D_{i+1} | D_i,\, D_{i-1}, ...,\, D_0) \\ &= \prod_{i=0}^{N-2} P(D_{i+1} | D_i,\, D_{i-1},\, D_{i-2},\, D_{i-3}) \end{aligned} \tag{9}$$

2 Derivation of $k(r, i_0, c)$

On each side of the square region R we can place

$$\begin{aligned} \epsilon &= 2c + 1 - i_0 - \underbrace{1}_{z_1} - \underbrace{2}_{\text{corner points}} \\ &= 2c - 2 - i_o \end{aligned}$$

points without the two corner points. For each number of points in region R, $r = \{2, 3, 4\}$, the number of possible configurations is counted (see Figure below for $r = 2$). The approach is analogous for $r = 3$ and $r = 4$.

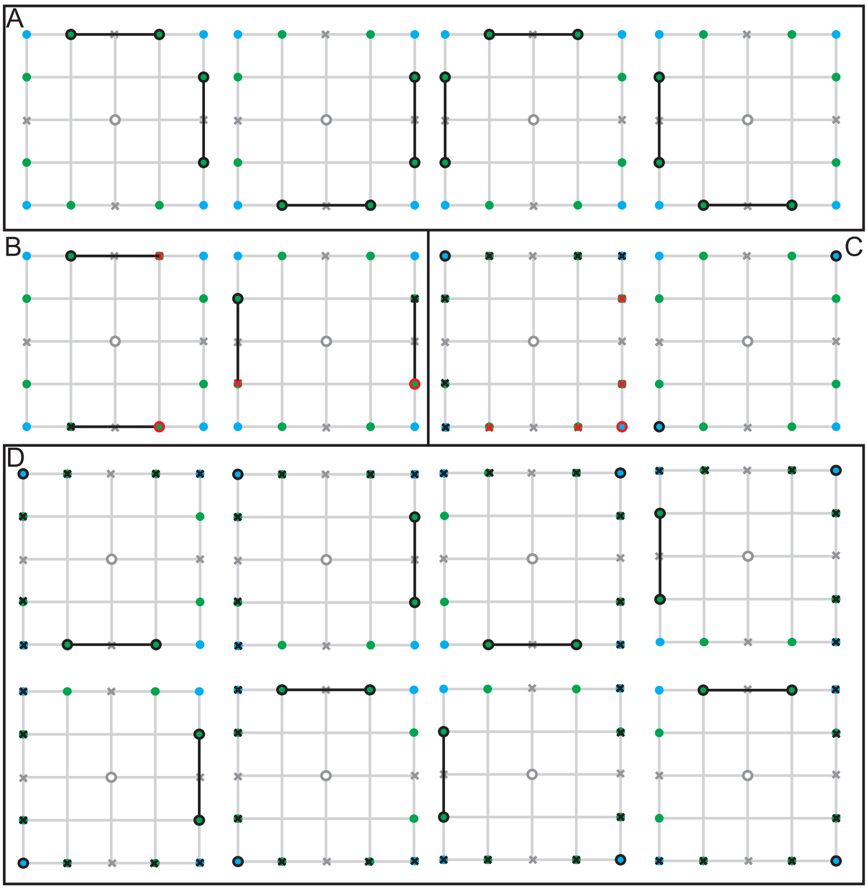

Counting the number of possible configuration of 2 points on region R. Let $\epsilon = 2c - i_0 - 2$, the number of free points on an edge of region R (without the corner points shown in blue and the possibilities excluded by z_1(at the center in Grey), shown as grey crosses). Here $i_0 = 0$ for convenience, thus $\epsilon = 2$ points (shown in green) **A**: One can choose any points from two adjacent edges. That means ϵ^2 possibilities to place two points and there are 4 such configurations ($= 4\epsilon^2$). **B**: When placing points on opposing edges, the placement of the first point forbids one possibility on the opposing edge (black cross for the black point and red cross for the red point). Thus there are $2\epsilon(\epsilon - 1)$ possible configurations. **C**: Placing one point on the corner forbids both edges connected by this corner (all points marked with a cross). The points thus have to be on opposite corners. There are only 2 possible configurations in this case **D**: Placing the first point on a corner leaves one of the two opposing edges to place points on. There are 8ϵ possibilities. In total for all cases there are $2\epsilon(\epsilon - 1) + 4\epsilon^2 + 8\epsilon + 2 = 6\epsilon^2 + 6\epsilon + 2$ possibilities.

3 Validation of the formula

To verify the correctness of our formulas, we calculated the exact distribution of $P(D_i = a)$ for $N = 7$ and $N = 8$ by generating all $N!$ possible point configurations and counting the relative frequency of each possible value for D_i. Then we compared the exact values to the values for $P(D_i = a)$ calculated from Formula 2 and found that they agree (Tables 1 and 2).

Additionally, we checked the validity of our formula for larger N ($N = 20$) by taking 10^6 random configurations and comparing the empirical frequency $h(d_i)$ with $P(d_i)$. The Mean

	1	2	3	1	2	3
1	0.6000	0.4000	0.0000	0.6000	0.4000	0.0000
2	0.0667	0.9333	0.0000	0.0667	0.9333	0.0000
3	0.0000	0.6000	0.4000	0.0000	0.6000	0.4000
4	0.0000	0.0667	0.9333	0.0000	0.0667	0.9333
5	0.0000	0.0000	1.0000	0.0000	0.0000	1.0000
6	0.0000	0.0000	1.0000	0.0000	0.0000	1.0000

Table 1: Exact probabilities (right) and $P(D_i = a)$ (left) for $N = 7$. The rows show the values of i and the columns the values of a.

	1	2	3	4	1	2	3	4
1	0.5238	0.4762	0.0000	0.0000	0.5238	0.4762	0.0000	0.0000
2	0.0476	0.8381	0.1143	0.0000	0.0476	0.8381	0.1143	0.0000
3	0.0000	0.3714	0.6286	0.0000	0.0000	0.3714	0.6286	0.0000
4	0.0000	0.0286	0.9714	0.0000	0.0000	0.0286	0.9714	0.0000
5	0.0000	0.0000	1.0000	0.0000	0.0000	0.0000	1.0000	0.0000
6	0.0000	0.0000	0.1429	0.8571	0.0000	0.0000	0.1429	0.8571
7	0.0000	0.0000	0.0000	1.0000	0.0000	0.0000	0.0000	1.0000

Table 2: Exact probabilities (right) and $P(D_i = a)$ (left) for $N = 8$. The rows show the values of i and the columns the values of a.

relative difference between $h(d_i)$ and $P(d_i)$ is 0.0001979655. The absolute differences in each cell are shown in Table 3.

4 Anderson-Darling

The theoretical and empirical cumulative distribution functions $F_i(c)$ and $E_i(c)$ are defined as follows:

$$F_i(c) = P(C_i \leq c) = \frac{1}{N}\sum_{z=1}^{N} P(D_i \leq c \mid D_{i-1} = d_{i-1}^z)$$

$$E_i(c) = \frac{1}{N}\sum_{z=1}^{N} \mathbf{I}[d_i^z \leq c]$$

E_i can be compared to F_i by an Anderson-Darling or a Cramér-von Mises test, which proved inferior to Pearson's χ^2 test described in the main manuscript (see Figure below).

	a									
	1	2	3	4	5	6	7	8	9	10
1	3.33E-05	9.24E-05	6.81E-05	6.67E-05	9.16E-06	0.00E+00	0.00E+00	0.00E+00	0.00E+00	0.00E+00
2	1.63E-05	4.47E-05	2.82E-05	5.07E-06	4.49E-06	4.49E-07	0.00E+00	0.00E+00	0.00E+00	0.00E+00
3	0.00E+00	1.01E-05	8.73E-05	1.55E-04	6.44E-05	7.29E-06	0.00E+00	0.00E+00	0.00E+00	0.00E+00
4	0.00E+00	4.45E-06	2.51E-06	2.37E-04	1.61E-04	6.87E-05	0.00E+00	0.00E+00	0.00E+00	0.00E+00
5	0.00E+00	0.00E+00	2.39E-05	3.59E-05	1.96E-04	2.56E-04	0.00E+00	0.00E+00	0.00E+00	0.00E+00
6	0.00E+00	0.00E+00	9.57E-07	1.82E-05	5.78E-05	7.17E-06	6.78E-05	0.00E+00	0.00E+00	0.00E+00
7	0.00E+00	0.00E+00	0.00E+00	1.01E-05	9.44E-06	1.22E-04	1.23E-04	0.00E+00	0.00E+00	0.00E+00
8	0.00E+00	0.00E+00	0.00E+00	6.19E-07	2.83E-05	9.83E-05	1.27E-04	0.00E+00	0.00E+00	0.00E+00
9	0.00E+00	0.00E+00	0.00E+00	0.00E+00	3.12E-06	7.83E-05	8.14E-05	0.00E+00	0.00E+00	0.00E+00
10	0.00E+00	0.00E+00	0.00E+00	0.00E+00	1.75E-07	5.47E-05	1.65E-04	2.20E-04	0.00E+00	0.00E+00
11	0.00E+00	0.00E+00	0.00E+00	0.00E+00	0.00E+00	5.49E-06	1.11E-04	1.16E-04	0.00E+00	0.00E+00
12	0.00E+00	0.00E+00	0.00E+00	0.00E+00	0.00E+00	1.65E-06	1.67E-05	1.51E-05	0.00E+00	0.00E+00
13	0.00E+00	0.00E+00	0.00E+00	0.00E+00	0.00E+00	0.00E+00	1.10E-05	1.10E-05	0.00E+00	0.00E+00
14	0.00E+00	0.00E+00	0.00E+00	0.00E+00	0.00E+00	0.00E+00	8.51E-07	1.20E-04	1.21E-04	0.00E+00
15	0.00E+00	0.00E+00	0.00E+00	0.00E+00	0.00E+00	0.00E+00	0.00E+00	2.45E-05	2.45E-05	0.00E+00
16	0.00E+00	0.00E+00	0.00E+00	0.00E+00	0.00E+00	0.00E+00	0.00E+00	1.24E-05	1.24E-05	0.00E+00
17	0.00E+00	0.00E+00	0.00E+00	0.00E+00	0.00E+00	0.00E+00	0.00E+00	0.00E+00	2.22E-15	0.00E+00
18	0.00E+00	0.00E+00	0.00E+00	0.00E+00	0.00E+00	0.00E+00	0.00E+00	0.00E+00	3.72E-06	3.72E-06
19	0.00E+00	0.00E+00	0.00E+00	0.00E+00	0.00E+00	0.00E+00	0.00E+00	0.00E+00	0.00E+00	0.00E+00

Table 3: Differences between the theoretical values of $P(d_i)$ and the empirical frequencies $h(d_i)$ from 10^6 samples. We sampled data with $N = 20$, a is in the columns of the table and runs form 1 to $\left\lfloor \frac{N}{2} \right\rfloor$, i is in the rows in goes from 1 to N.

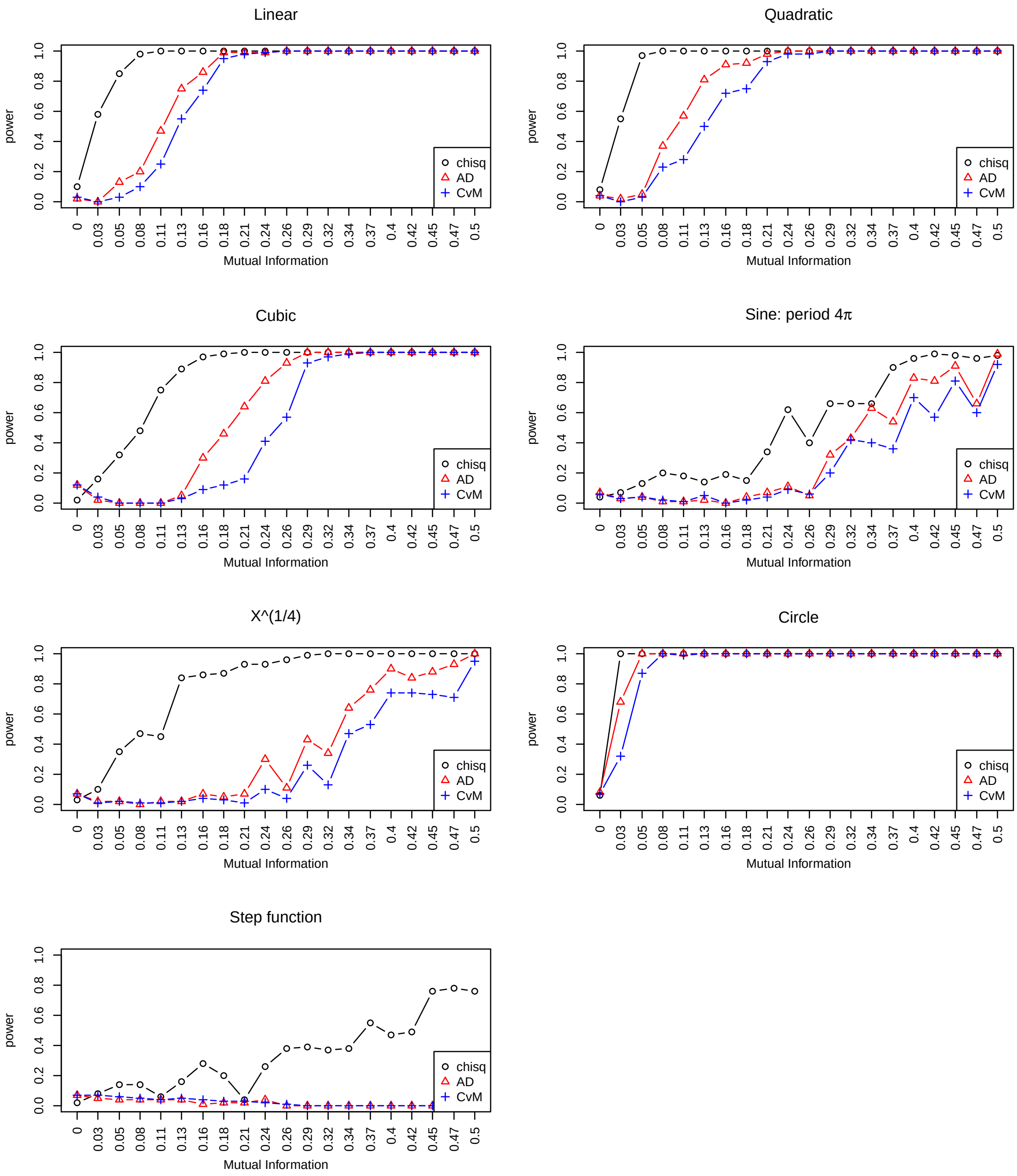

Benchmark comparing Pearson's χ^2 test as novel distributional test based on the theoretical and empirical probability functions (black curve labeled chisq) against using an Anderson-Darling (red curve, labeled AD) or a Cramér-von Mises test (blue curve labeled CvM) on the cumulative distribution functions.

5 Noise levels for all functional dependencies

MI	lin	para	quadratic	sin1	sin2	circ	x14	step
0.001	6.39	6.59	15.88	15.64	15.64	6.39	3.60	11.05
0.027	1.21	1.25	2.71	2.97	2.97	1.21	0.69	2.10
0.054	0.86	0.88	1.79	2.10	2.10	0.86	0.48	1.48
0.08	0.69	0.71	1.37	1.70	1.70	0.69	0.39	1.20
0.106	0.59	0.61	1.11	1.45	1.45	0.59	0.33	1.02
0.132	0.52	0.54	0.93	1.28	1.28	0.52	0.29	0.91
0.159	0.47	0.49	0.79	1.15	1.16	0.47	0.26	0.81
0.185	0.43	0.44	0.68	1.05	1.05	0.43	0.24	0.74
0.211	0.40	0.41	0.59	0.97	0.97	0.40	0.22	0.69
0.237	0.37	0.38	0.52	0.90	0.90	0.37	0.21	0.64
0.264	0.35	0.35	0.45	0.84	0.84	0.35	0.19	0.59
0.29	0.32	0.33	0.39	0.79	0.79	0.32	0.18	0.56
0.316	0.31	0.31	0.34	0.75	0.75	0.31	0.17	0.52
0.342	0.29	0.30	0.30	0.71	0.71	0.29	0.16	0.49
0.369	0.27	0.28	0.25	0.67	0.67	0.27	0.15	0.47
0.395	0.26	0.26	0.22	0.64	0.64	0.26	0.14	0.44
0.421	0.25	0.25	0.18	0.61	0.60	0.25	0.14	0.42
0.447	0.24	0.24	0.15	0.58	0.58	0.24	0.13	0.40
0.474	0.23	0.23	0.12	0.55	0.55	0.23	0.12	0.37
0.5	0.22	0.22	0.09	0.53	0.53	0.22	0.12	0.35

All noise levels generated from the target mutual information values between 0.001 and 0.5 shown in the first column. Each column starting from the second represents a functional dependency in the order: linear, quadratic, cubic, sine with period 4π, sine with period 16π, circular, $x^{1/4}$ and step function and gives the 20 different noise levels used in the benchmark for each case

6 Example usage of CRAN package

```
library(knnIndep)
colnames(noises) = 1:2
colnames(noises) = c("1",".2") #mutual information of the noise levels
vals = run.tests(novelTest.extreme, list(maxi=30),1:2,noises,100)
power.cor = drop(calculate.power(vals))
plot.power(list(cor=power.cor),t(noises))
```
